# Co-Enzyme Q10 and *n*-3 Polyunsaturated Fatty Acid Supplementation Reverse Intermittent Hypoxia-Induced Growth Restriction and Improved Antioxidant Profiles in Neonatal Rats

**DOI:** 10.3390/antiox6040103

**Published:** 2017-12-16

**Authors:** Kay D. Beharry, Charles L. Cai, Michael M. Henry, Sara Chowdhury, Gloria B. Valencia, Jacob V. Aranda

**Affiliations:** 1 Department of Pediatrics, Division of Neonatal-Perinatal Medicine, State University of New York, Downstate Medical Center, Brooklyn, NY 11203, USA; charles.cai@downstate.edu (C.L.C.); michael.henry@downstate.edu (M.M.H.); Sara.chowdhury@downstate.edu (S.C.); Gloria.valencia@downstate.edu (G.B.V.); jacob.aranda@downstate.edu (J.V.A.); 2 Department of Ophthalmology; State University of New York, Downstate Medical Center, Brooklyn, NY 11203, USA; 3 SUNY Eye Institute, State University of New York, New York, NY 10062, USA

**Keywords:** antioxidants, coenzyme Q10, growth factors, *n*-3 polyunsaturated fatty acids, Neonatal Intermittent Hypoxia, oxidative stress, postnatal growth

## Abstract

Neonatal intermittent hypoxia (IH) increases the risk for many morbidities in extremely low birth weight/gestational age (ELBW/ELGA) neonates with compromised antioxidant systems and poor growth. We hypothesized that supplementation with coenzyme Q10 (CoQ10, ubiquinol) or *n*-3 polyunsaturated fatty acids (PUFAs) during neonatal IH improves antioxidant profiles and somatic growth in neonatal rats. Newborn rats were exposed to two IH paradigms at birth (P0): (1) 50% O_2_ with brief hypoxic episodes (12% O_2_); or (2) room air (RA) with brief hypoxia, until P14 during which they received daily oral CoQ10 in olive oil, *n*-3 PUFAs in fish oil, or olive oil only from P0 to P14. Pups were studied at P14 or placed in RA until P21 for recovery from IH (IHR). Body weight and length; organ weights; and serum antioxidants and growth factors were determined at P14 and P21. Neonatal IH resulted in sustained reductions in somatic growth, an effect that was reversed with *n*-3 PUFAs. Improved growth was associated with higher serum growth factors. CoQ10 decreased superoxide dismutase (SOD) and glutathione, but increased catalase, suggesting reduced oxidative stress. Further studies are needed to determine the synergistic effects of CoQ10 and *n*-3 PUFA co-administration for the prevention of IH-induced oxidative stress and postnatal growth deficits.

## 1. Introduction

Neonatal intermittent hypoxia (IH), defined as brief, repetitive cycles of arterial oxygen desaturations followed by re-oxygenation, often occurs in extremely low birth weight (ELBW)/extremely low gestational age (ELGA) neonates requiring oxygen supplementation and/or mechanical ventilation [1]. An IH event is usually defined as a decline in SaO_2_ by 5% lasting <3 min in duration [2,3,4,5]. Re-oxygenation following an IH event (IHR) can occur in normoxia or hyperoxia, but whether the effects of IH with resolution in normoxia are less deleterious than that in hyperoxia, remains to be determined. Re-oxygenation induces damaging mitochondrial reactive oxygen species (ROS), which not only causes oxidative stress and injury, but also activates signaling mechanisms to counteract those induced by IH. After numerous episodes, occurring within minutes of each other, a “critical” point is achieved where the mechanisms induced by IH become indistinguishable from those induced by IHR [6]. The merging of these mechanisms has profound deleterious effects on mitochondrial homeostasis and redox state [7]. 

IH-induced ROS are toxic and damaging to lipid cell membranes leading to lipid peroxidation [8]. In addition, ROS destroy cell enzyme mechanisms and DNA; it plays a major role in inflammatory processes, and impairment of multiple neonatal systems, including the brain [9], eyes [6,10], heart [11] lungs [5,12], liver [13,14], and kidneys [15,16]. ROS are implicated in the pathogenesis, progression, and severity of a variety of neonatal morbidities, such as retinopathy of prematurity, chronic lung disease, patent ductus arteriosus, apnea of prematurity/bradycardia, sepsis, intraventricular hemorrhage [17,18,19,20]. These are referred to as “oxygen radical diseases of neonatology” [21,22]. ELBW/ELGA neonates are particularly susceptible to ROS accumulation and damage due to compromised antioxidant systems [23,24,25]. The principal ROS is superoxide anion which is rapidly dismutated by superoxide dismutase (SOD), to the more stable hydrogen peroxide (H_2_O_2_) and O_2_ [26]. Catalase and glutathione scavenge H_2_O_2_ and prevent its accumulation. Since free iron is an important catalyst for ROS reactions [27], excessive H_2_O_2_ will react with iron to form the highly reactive hydroxyl radical in the Haber–Weiss reaction that causes lipid peroxidation [18]. ELBW/ELGA neonates are often supplemented with iron during the early neonatal period and their low levels of plasma transferrin, the major iron-binding protein [28], predispose them to a higher risk for lipid peroxidation [8].

ELBW/ELGA neonates experiencing the highest incidence of IH are more vulnerable to malnutrition, extrauterine growth restriction, and nutrient deficits. This is largely due to withholding of enteral and parenteral nutrition as these infants are unable to coordinate sucking, swallowing, and breathing. One study of close to 600 preterm infants examining 300,000 desaturations (oxygen saturation, SpO_2_ ≤ 80%) and 100,000 bradycardia (heart rate ≤ 80 beats per minute alarms showed a correlation with the timing and frequency of the enteral feeding [29]. ELBW/ELGA neonates receiving parenteral soybean or olive oil-based lipid nutrition and even enteral nutrition often develop deficits in *n*-3 polyunsatuarated fatty acids (PUFAs) such as docosahexaenoic acid (DHA) [30,31,32]. Supplementation with the *n*-3 PUFAs has been shown to reduce inflammation and reduce the severity of major neonatal diseases, presumably due to its antioxidant properties [33,34,35]. 

Coenzyme Q10 (CoQ10) plays a key role in energy production and acts as an electron-shuttling compound. It shuttles electrons from complexes I and II to complex III [36,37]. Therefore, CoQ10 is vital to the mitochondrial electron transport chain and has potent antioxidant properties. CoQ10 is fat soluble and is found in tissues with high energy demands such as the brain, eyes, liver, and kidneys [38]. Numerous studies have shown the benefits of CoQ10 including reversing mitochondrial dysfunction and increasing cellular metabolism [39,40,41]. CoQ10 is involved in the prevention of membrane phospholipid peroxidation and free radical oxidation [42], and has been shown to protect erythrocytes of preterm infants from hemolysis by H_2_O_2_ [43]. Co-Q10 deficiency in newborns is associated with fatal neonatal multi-organ failure which may resolve with early CoQ10 supplementation [44]. The process of childbirth which involves movement from a hypoxic intrauterine environment to the extrauterine environment, increases oxidative stress and ROS production, a process called “oxidative aggression” [45]. In term infants, oxidative aggression is counteracted by antioxidants transferred from the mother. However, in preterm infants with a shortened gestational period interrupting maternal transfer of antioxidants, the defenses against oxidative aggression is reduced, making them more vulnerable to lipid peroxidation and oxygen free radical diseases of the neonate [21,22]. Studies have shown lower Co-Q10 amniotic fluid levels in preterm pregnancies [46], and higher biomarkers of lipid peroxidation, co-incident with lower antioxidant plasma levels in preterm infants compared to term infants, reflecting greater oxidative stress [45]. Despite the numerous reports of the beneficial effects of *n*-3 PUFA and CoQ10 supplementation on growth and nutrition as well as oxidative stress, no previous studies have examined the effects of their supplementation for improving IH-induced growth restriction and antioxidant profiles. We therefore conducted a series of experiments to test the hypothesis that supplementation with *n*-3 PUFAs or CoQ10 reverses IH-induced growth restriction by altering factors that influence postnatal growth, and improves the antioxidant profiles of newborn rats. To prove our hypothesis, we employed two different, but relevant IH paradigms: (1) exposure to hyperoxia (50% O_2_) with brief 1-min hypoxia (12% O_2_) events; or (2) exposure to atmospheric oxygen with brief 1-min hypoxia (12% O_2_) events during which animals were supplemented with either CoQ10, *n*-3 PUFAs, or olive oil (controls). Our hypothesis was tested with the following objectives: (1) to examine and compare the effects of CoQ10 and *n*-3 PUFA supplementation during IH exposure on somatic growth accretion in neonatal rats; (2) to establish whether CoQ10 and/or *n*-3 PUFAs influence factors that regulate postnatal growth; and (3) to determine whether CoQ10 and/or *n*-3 PUFAs improve the antioxidant profiles of neonatal rats exposed to IH. These studies of different treatment strategies and IH paradigms, extend our previous work [6,12,13,15,23,47], and therefore describe a more complete picture of the heterogeneity in responses during IH that are clinically relevant since IH events experienced by ELBW/ELGA neonates can resolve in normoxia or hyperoxia. 

## 2. Material and Methods

All experiments were approved by the State University of New York, Downstate Medical Center Institutional Animal Care and Use Committee, Brooklyn, NY, USA. The ethical approval number is 17-10517. Animals were treated humanely, according to the guidelines outlined by the United States Department of Agriculture and the Guide for the Care and Use of Laboratory Animals. 

### 2.1. Experimental Design

Certified infection-free, timed-pregnant Sprague Dawley rats were purchased from Charles River Laboratories (Wilmington, MA, USA) at 18 days gestation. The animals were housed in an animal facility with a 12-h-day/12-h-night cycle and provided standard laboratory diet and water ad libitum until delivery. Within 2–4 h of birth, newborn rat pups delivering on the same day were pooled and randomly assigned to expanded litters of 18 pups/litter (9 males and 9 females). Gender was identified by the anogenital distance. The expanded litter size was used to simulate relative postnatal malnutrition of ELBW/ELGA neonates who are at increased risk for oxidative stress. Animals were exposed to neonatal IH from P0 to P14 (IH) or allowed to recover in room air (RA) until P21 (IHR). RA littermates were raised in atmospheric oxygen from P0 to P14 or P0 to P21, and served as controls. During IH (P0 to P14), pups were administered daily oral doses of CoQ10 (0.35 mg in 50 µL extra virgin olive oil) purchased from Sigma Aldrich (St. Louis, MO, USA), 50 µL fish oil containing 35 mg total *n*-3 PUFAs (22 mg eicosapentaenoic acid (EPA); and 13 mg docosahexaenoic acid (DHA)), or 50 µL extra virgin olive oil. Pups were euthanized at P14 or allowed to recover from IH in RA from P14 to P21 (IHR). Total body weight (grams) and linear growth (crown to rump length, cm) were recorded at P0, P14, and P21 to determine percentage change and weight accretion from birth. Weight accretion evidenced by percentage changes in body weight and linear growth were calculated as weight or length at the end of the experiment (P14 or P21) minus weight or length at birth (P0) divided by the weight or length at birth × 100. Percentage change in body weight was used to standardize differences in birth weight.

### 2.2. Neonatal Intermittent Hypoxia (IH) Profiles

**Figure 1:**
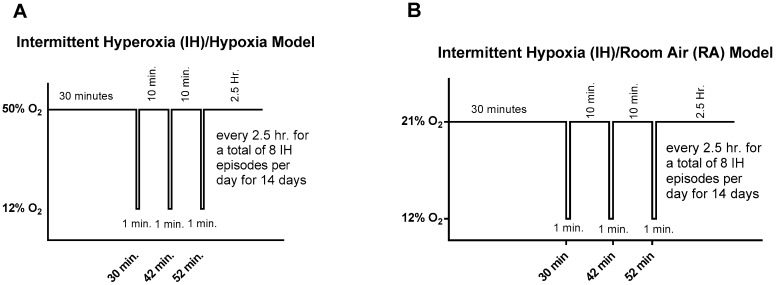
Representation of the two neonatal intermittent hypoxia (IH) paradigms used in this study. Hyperoxia (50% O_2_) following a brief, repetitive hypoxic event (12% O_2_) is presented in (**A**). Normoxia (21% O_2_) following a brief, repetitive hypoxic event (12% O_2_) is presented in (**B**).

Episodes of re-oxygenation following an IH event may occur in normoxia or hyperoxia. We therefore employed two IH paradigms in these experiments to assess whether re-oxygenation in normoxia between each IH episode results in less adverse outcomes than re-oxygenation in hyperoxia (50% O_2_) between IH episodes. Examples of the two IH paradigms are presented in Figure 1. Animals randomized to IH were placed with the dams in specialized oxygen chambers (BioSpherix, NY, USA) attached to an oxycycler. These chambers were optimized for gas efficiency and provided adequate ventilation for the animals in a controlled atmosphere with minimal gas usage. Oxygen content inside the chamber was continuously monitored and recorded on a Dell Computer. Carbon dioxide in the chamber was monitored and removed from the atmosphere by placing soda lime within the chamber. The IH profiles consisted of: (1) an initial exposure of hyperoxia (50% O_2_) for 30 min followed by three brief, 1-min, clustered hypoxic events (12% O_2_), with a 10-min re-oxygenation in 50% O_2_ between each hypoxic event. Recovery from IH (IHR) occurred in 50% O_2_ following each clustered IH event for 2.5 h for a total of 8 clustering IH episodes per day for 14 days, as previously described [6,12,13,15,47]; or (2) an initial exposure of normoxia (21% O_2_) for 30 min followed by three brief, 1-min, clustered hypoxic events (12% O_2_), with a 10-min re-oxygenation in 21% O_2_ between each hypoxic event. Recovery occurred in normoxia following each clustered IH event for 2.5 h for a total of 8 clustering IH episodes per day for 14 days. CoQ10, *n*-3 s, or olive oil supplementation occurred only during IH from P0 to P14 and not during IHR (P14–P21). Animals were either euthanized immediately following IH on P14, or placed in RA for 7 days recovery, then euthanized at P21. Oxygen saturation was confirmed on a sentinel unanesthetized rat pup from each group using the MouseOx Pulse Oximeter and WinDaq Waveform Browser software (STARR Life Sciences Corp., Oakmont, PA, USA) before and after IH exposure. This procedure was conducted to confirm that the animals were hypoxic during IH cycling.

### 2.3. Sample Collection and Processing

At euthanasia, mixed arterial and venous blood samples were collected following decapitation, into sterile Eppendorf tubes and placed on ice for 30 min. Samples were centrifuged at 3500 rpm for 30 min at 4 °C. The resulting serum was transferred to a clean sterile Eppendorf tube and frozen at −20 °C until analysis. All samples were analyzed on the same day. A total of 8 serum samples per group (4 males and 4 females) were analyzed. 

### 2.4. Assay of Serum Antioxidants

Serum levels of SOD, catalase and total glutathione were determined using commercially available activity assay kits purchased from Cayman Chemical (Ann Arbor, MI, USA). Samples were processed and assayed according to the manufacturer’s protocol. 

### 2.5. Assay of Serum Growth Hormone (GH) and Growth Hormone Receptor (GHR)

GH and GHR levels in the serum samples were determined using commercially-available rat GH, and GHR enzyme-linked immunosorbent assay (ELISA) kits purchased from MyBioSource (San Diego, CA, USA). Samples were processed and assayed according to the manufacturer’s protocol. 

### 2.6. Assay of Serum Insulin-Like Growth Factor (IGF)-I and Insulin-Like Growth Factor Binding Proteins (IGFBPs)

Serum IGF-I and IGFBP levels were determined using commercially-available rat IGF-I Quantikine ELISA kits and mouse IGFBP-1 and IGFBP-3 duoset ELISA kits purchased from R&D Systems (Minneapolis, MN, USA), following a pre-treatment step to separate serum IGF-I from its binding proteins. All samples were processed and assayed according to the manufacturer’s protocol and the mouse duoset IGFBP ELISA kits detected rat IGFBPs. 

### 2.7. Assay of Serum Vascular Endothelial Growth Factor (VEGF) and Soluble Vascular Endothelial Growth Factor Receptor (sVEGFR-1)

Serum VEGF and sVEGFR-1 levels were determined using commercially-available rat VEGF and mouse VEGFR-1 Quantikine ELISA kits, respectively, purchased from R&D Systems (Minneapolis, MN, USA). All samples were processed and assayed according to the manufacturer’s protocol and the mouse VEGFR-1 ELISA kits detected rat VEGFR-1. 

### 2.8. Statistical Analysis

To determine differences among the RA and two IH groups, a test for normality was first conducted using the Bartlett’s test. Normally distributed data were analyzed using two-way analysis of variance (ANOVA) with Dunnett’s multiple comparison post-hoc tests. Non-normally distributed data were analyzed using Kruskall–Wallis test with Dunn’s multiple comparison test. Similar analyses were conducted to determine differences among the treated groups within each oxygen environment. Data are presented as mean ± SD and a *p*-value of <0.05 was considered as statistically significant using Statistical Package for Social Sciences (SPSS) version 16.0 (SPSS Inc., Chicago, IL, USA). Graphs were prepared using GraphPad Prizm version 7.03 (GraphPad, San Diego, CA, USA).

## 3. Results

### 3.1. Effects on Somatic Growth

**Figure 2:**
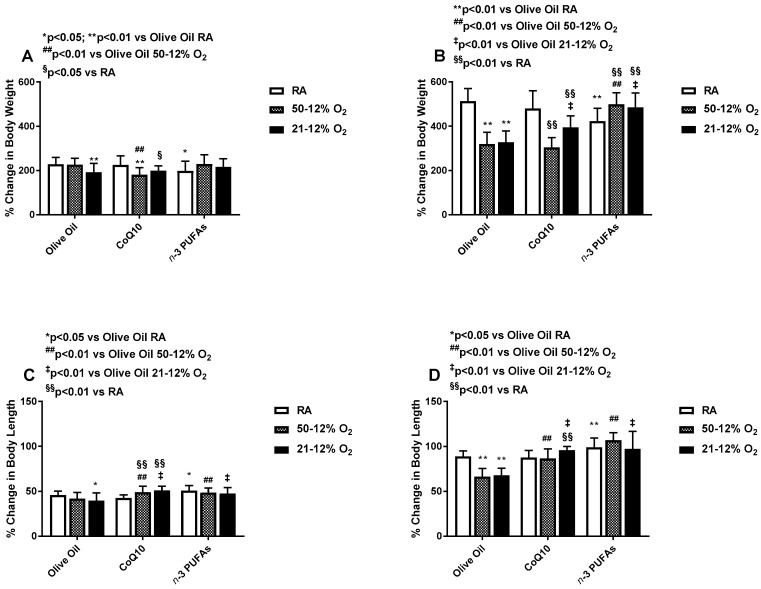
Effects of neonatal intermittent hypoxia (IH) with Co-Q10 or *n*-3 polyunsaturated fatty acids (PUFA) supplementation on percentage change in body weight panels (**A**,**B**), and linear growth panels (**C**,**D**). Panels (**A**,**C**) represent treatment during IH exposure and supplementation for 14 days, and panels (**B**,**D**) represent recovery from IH (IHR) in room air (RA) with no treatment. The white bar represents normoxia control animals exposed to RA only. These animals were raised in atmospheric oxygen from birth to P14 or P21. The hashed bar represents animals exposed to 50–12% O_2_, and the solid black bar represents animals exposed to 21–12% O_2_. Data are expressed as mean ± SD (*n* = 8 samples/group).

Percentage changes in body weight and length at P14 (IH) and P21 (IHR) are presented in Figure 2. Data showed that exposure to IH reduced weight accretion during olive oil and CoQ10 supplementation compared to RA. In contrast, weight accretion was increased in the IH groups during *n*-3 PUFA supplementation (Figure 2A). During the recovery period, weight accretion remained significantly suppressed in the IH groups supplemented with olive oil and CoQ10, compared to RA. However, weight accretion in the IH groups supplemented with *n*-3 PUFAs surpassed their RA littermates and were comparable with the RA groups treated with olive oil (Figure 2B). Overall, supplementation with *n*-3 PUFAs in RA resulted in lower body weight accretion compared to supplementation with olive oil and CoQ10 in RA. However, the suppressive effects of IH on body weight accretion were reversed with *n*-3 PUFAs during IH and IHR. Compared to RA, exposure to 21–12% O_2_ had negative effects on linear growth in the groups supplemented with olive oil (Figure 2C). These suppressive effects remained sustained during IHR in both IH groups (Figure 2D). Conversely, CoQ10 and *n*-3 PUFAs increased body length in IH (Figure 2C, but the effect was more robust during IHR (Figure 2D). Table 1 shows that compared to olive oil, treatment with CoQ10 increased kidney/body weight ratios in RA and brain/body weight ratios in 50–12% O_2_, but reduced liver/body weight ratios in both IH groups, compared to olive oil. *n*-3 PUFAs increased brain/body and kidney/body weight ratios in RA, and liver/body weight ratios in both IH groups. *n*-3 PUFAs had no suppressive effects on organ weights during treatment in IH. Table 2 lists the organ to body weight ratios during IHR and post treatment at P21. Compared to olive oil, CoQ10 and *n*-3 PUFA supplementation increased liver/body weight ratios in RA, while CoQ10 reduced it in 21–12% O_2_. *n*-3 PUFA supplementation increased liver/body weight ratios in all oxygen environment as well as brain/body and kidney/body weight ratios in RA during the IHR period. 

### 3.2. Effects on Serum GH and GHR

**Figure 3:**
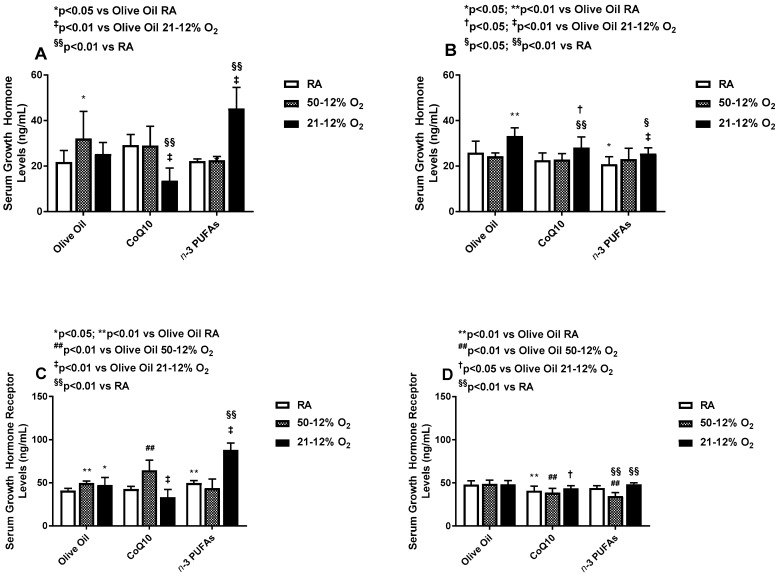
Effects of neonatal IH with Co-Q10 or *n*-3 PUFA supplementation on serum growth hormone panels (**A**,**B**) and growth hormone receptor panels (**C**,**D**) levels. Groups are as described in Figure 2. Data are expressed as mean ± SD (*n* = 8 samples/group).

During treatment, serum GH levels were decreased in the 21–12% O_2_ group treated with Co-Q10 compared to RA and olive oil, and elevated in the 21–12% O_2_ group treated with *n*-3 PUFAs compared to RA and olive oil (Figure 3A). During IHR, GH levels were highest in the groups exposed to 21–12% O_2_ IH compared to RA, regardless of treatment, but rem ained higher with CoQ10 and *n*-3 PUFA supplementation (Figure 3B). Compared to olive oil treatment in RA, GH levels declined with *n*-3 PUFA supplementation in RA (Figure 3B). Serum GHR levels mirrored those of GH during treatment in IH (Figure 3C) and in IHR post treatment (Figure 3D), but to a lesser degree.

### 3.3. Effects on Serum IGF and IGFBPs

**Figure 4:**
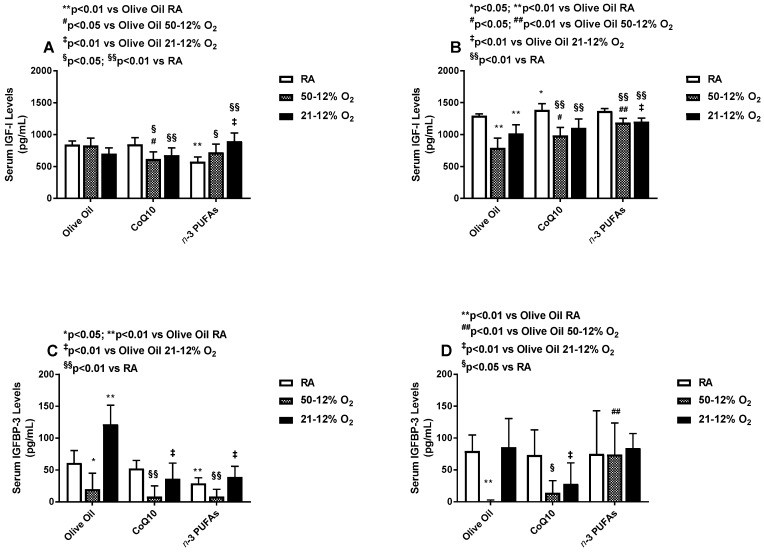
Effects of neonatal IH with Co-Q10 or *n*-3 PUFA supplementation on serum insulin-like growth factor-I panels (**A**,**B**) and insulin-like growth factor binding protein-3 panels (**C**,**D**) levels. Groups are as described in Figure 2. Data are expressed as mean ± SD (*n* = 8 samples/group).

**Figure 5:**
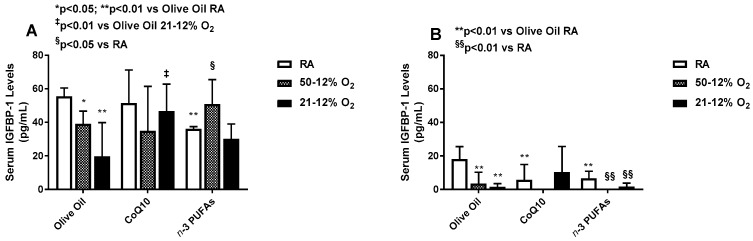
Effects of neonatal IH with Co-Q10 or *n*-3 PUFA supplementation on serum insulin-like growth factor binding protein-1 levels. Panel (**A**) represents treatment during IH exposure and supplementation for 14 days, and panel (**B**) represents recovery from IH (IHR) in room air (RA) with no treatment. Groups are as described in Figure 2. Data are expressed as mean ± SD (*n* = 8 samples/group).

Supplementation with *n*-3 PUFAs in RA, but not IH, resulted in lower serum IGF-I levels (Figure 4A). CoQ10 decreased serum IGF-I in 50–12% O_2_ compared to olive oil and treatment in RA, while treatment in 21–12% O_2_ did not differ from olive oil, but was lower than treatment in RA (Figure 4A). *n*-3 PUFA treatment elevated serum IGF-I levels in the IH groups compared to RA, but lowered it in the RA group compared to olive oil (Figure 4A). During IHR post treatment, levels of IGF declined in all supplemented group exposed to IH compared to their RA counterparts, but supplementation with *n*-3 PUFAs resulted in higher serum IGF-I in both IH groups, compared to the RA controls (Figure 4B). Both CoQ10 and *n*-3 PUFAs decreased serum IGFBP-3 in all oxygen environments compared to RA. Exposure to 50–12% O_2_ resulted in major reductions in serum IGFBP-3 levels, while exposure to 21–12% O_2_ caused robust elevations with olive oil supplementation compared to RA as well as the CoQ10 and *n*-3 PUFAs littermates (Figure 4C). During IHR, the levels of IGFBP-3 further declined with olive oil supplementation in 50–12% O_2_, and with CoQ10 treatment in both IH paradigms compared to RA. Supplementation with *n*-3 PUFAs preserved serum IGFBP-3 levels during IHR in all oxygen environments (Figure 4D). During treatment in IH, serum IGFBP-1 levels declined with *n*-3 PUFAs in RA compared to olive oil, and with olive oil treatment in both IH paradigms compared to RA (Figure 5A). Treatment with CoQ10 increased serum IGFBP-1 in 21–12% O_2_, while the levels were elevated with and *n*-3 PUFAs in 50–12% O_2_ compared to RA (Figure 5A). During IHR post treatment, serum IGFBP-1 levels declined significantly in all groups. In RA, treatment with CoQ10 and *n*-3 PUFAs resulted in lower IGFBP-1 levels compared to olive oil, in 50–12% O_2_, CoQ10 and *n*-3 PUFAs abolished serum IGFBP-1 levels, and in 21–12% O_2_, CoQ10 non-significantly elevated serum IGFBP-1 levels (Figure 5B).

### 3.4. Effects on Serum VEGF and sVEGFR-1

**Figure 6:**
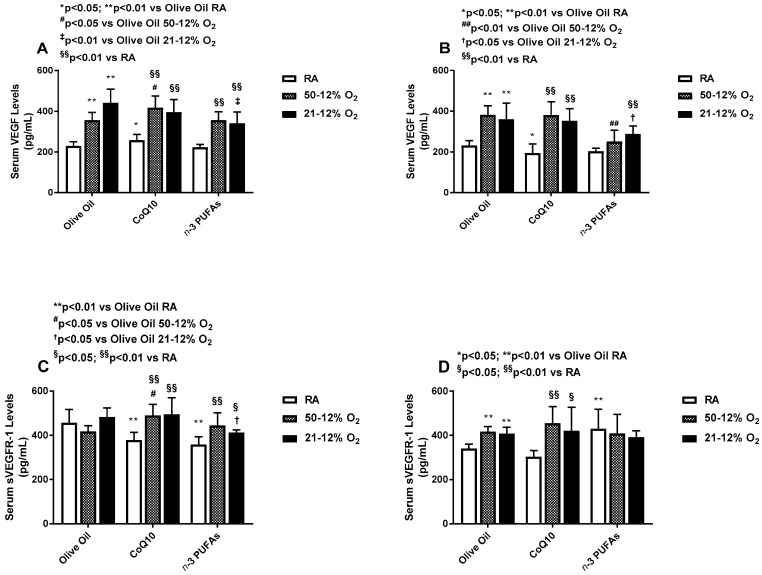
Effects of neonatal IH with Co-Q10 or *n*-3 PUFA supplementation on serum vascular endothelial growth factor panels (**A**,**B**) and soluble vascular endothelial growth factor receptor-1 panels (**C**,**D**) levels. Groups are as described in Figure 2. Data are expressed as mean ± SD (*n* = 8 samples/group).

Serum VEGF levels increased significantly during all treatments in both IH paradigms (Figure 6A) compared to RA, and the effect remained sustained during IHR post treatment although *n*-3 PUFA treatment in both IH groups resulted in much lower levels compared to RA and olive oil (Figure 6B). sVEGFR-1 levels increased with CoQ10 and *n*-3 PUFA supplementation in the IH groups compared to their RA counterparts (Figure 6C). In IHR post treatment, *n*-3 PUFA supplementation in RA caused a significant elevation in serum sVEGFR-1 compared to olive oil while the levels remained elevated in both IH groups supplemented with olive oil and CoQ10 compared to RA (Figure 6D). 

### 3.5. Effects on Serum Antioxidants

**Figure 7:**
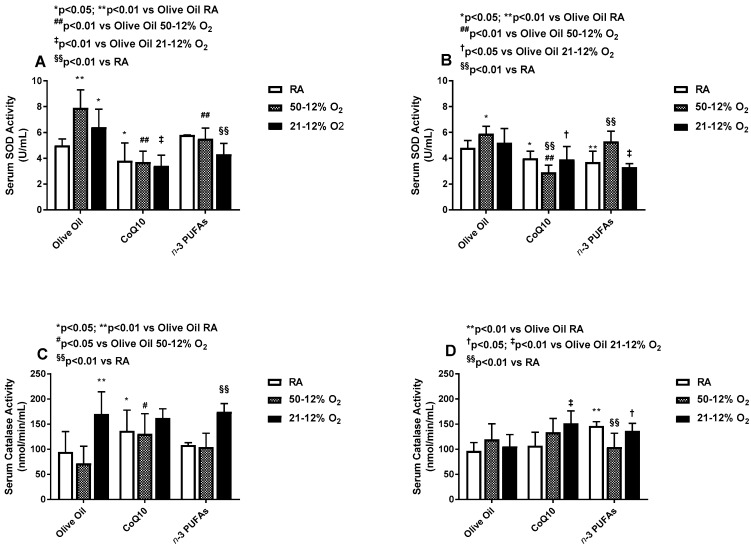
Effects of neonatal IH with Co-Q10 or *n*-3 PUFA supplementation on serum superoxide dismutase panels (**A**,**B**) and catalase panels (**C**,**D**) activities. Groups are as described in Figure 2. Data are expressed as mean ± SD (*n* = 8 samples/group).

**Figure 8:**
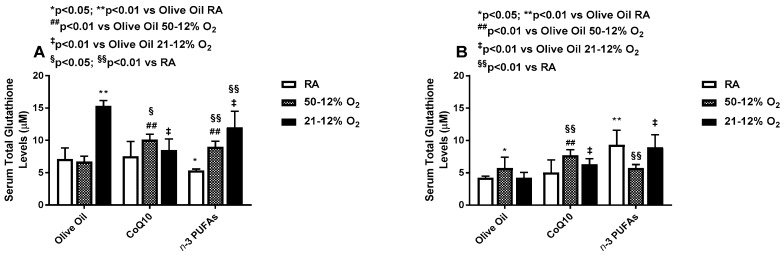
Effects of neonatal IH with Co-Q10 or *n*-3 PUFA supplementation on serum total glutathione levels. Panel (**A**) represents treatment during IH exposure and supplementation for 14 days, and panel (**B**) represents recovery from IH (IHR) in room air (RA) with no treatment. Groups are as described in Figure 2. Data are expressed as mean ± SD (*n* = 8 samples/group).

Serum SOD activity was increased in both IH groups supplemented with olive oil, compared to the RA. Supplementation with CoQ10 suppressed SOD activity in all groups, while supplementation with *n*-3 PUFAs reduced it in 21–12% O_2_ compared to olive oil, and in 50–12% O_2_ compared to RA (Figure 7A). During IHR, SOD activity remained elevated in the olive oil groups exposed to 50–12% O_2_ compared to RA and suppressed in the CoQ10 supplemented groups compared to RA and olive oil. There was a latent suppression of SOD activity in the *n*-3 PUFA groups supplemented in RA and 21–12% O_2_ compared to olive oil, but not 50–12 O_2_ (Figure 7B). Serum catalase activity was elevated in all 21–12% O_2_ groups compared to RA, but the most robust elevations were noted with olive oil and *n*-3 PUFAs. CoQ10 increased catalase activity in RA and 50–12% O_2_ compared to olive oil, but not 21–12% O_2_ (Figure 7C). This response pattern remained sustained during IHR, despite overall lower levels. Treatment with *n*-3 PUFAs in RA resulted in the highest serum catalase levels during IHR. Both CoQ10 and n-3 PUFA supplementation increased catalase activity in 21–12% O_2_, while *n*-3 PUFA supplementation reduced catalase activity in 50–12% O_2_ during IHR compared to RA and olive oil (Figure 7D). During IH, total serum glutathione was lowest in the RA group supplemented with *n*-3 PUFAs. In 50–12% O_2_, both CoQ10 and *n*-3 PUFAs increased total glutathione compared to olive oil and their RA littermates. In the 21–12% O_2_ groups, total glutathione was highest in the olive oil groups, consistent with the catalase response. This effect was suppressed with CoQ10 and *n*-3 PUFAs, although CoQ10 resulted in a much stronger suppressive effect (Figure 8A). During IHR post treatment, total glutathione levels were highest in the RA and 21–12% O_2_ groups supplemented with *n*-3 PUFAs compared to olive oil, while the levels were highest in the 50–12% O_2_ groups supplemented with CoQ10 (Figure 8B).

## 4. Discussion

### 4.1. Somatic Growth

This study found that supplementation with CoQ10 or *n*-3 PUFAs reduces IH-induced oxidative stress and reverses IH-induced growth restriction by improving antioxidant profiles and altering factors that influence postnatal growth. We employed two IH paradigms to compare re-oxygenation with RA to re-oxygenation in hyperoxia following an IH event as these two paradigms are clinically relevant. These studies provided several interesting and important findings regarding growth: (1) Re-oxygenation in RA following an IH event causes sustained deficits in body weight and length accretion comparable with re-oxygenation in hyperoxia following an IH event, with no evidence of catchup growth during the recovery, IHR period. This finding was surprising considering the shorter range of oxygen variation from 21% O_2_ to 12% O_2_ versus 50% O_2_ to 12% O_2_, and implicates IH, but not hyperoxia, in the sustained suppression of somatic growth; (2) Supplementation with CoQ10 did not reverse IH-induced growth restriction in the 50–12% O_2_ group, and moderately improved growth accretion in the 21–12% O_2_ group, but was effective for improving body length accretion in both IH groups; (3) Supplementation with *n*-3 PUFAs facilitated the most beneficial effects with significant improvements in body weight and length during and post treatment in IH, resulting in taller pups compared to their IH counterparts. This response is indicative of true overall growth and not catch-up fat which is characterized by weight gain and short stature. The findings of IH-induced growth restriction corroborate those of others [11]. It was interesting to note that in addition to overall body growth benefits, *n*-3 PUFA supplementation induced brain, liver and kidney mass and size suggesting that both CoQ10 and *n*-3 PUFAs may specifically target these organs with significant weight sparing in the case of *n*-3 PUFAs, particularly the liver when exposed to chronic neonatal IH. These beneficial effects of *n*-3 PUFAs on the liver and kidneys have been previously described [48,49,50]. However, this is the first report focusing on neonatal IH and suggests that ELBW/ELGA neonates who are nutritionally deprived, growth restricted, and who experience frequent IH episodes during a critical time of development may benefit substantially from *n*-3 PUFA supplementation and may partly explain the poor long-term outcome in some of these ELBW/ELGA neonates. 

### 4.2. Growth Factors

Neonatal IH is often associated with feeding intolerance, and poor growth and nutrition, and several studies in neonatal animal models have reported that IH is associated with significant growth restriction [11,51]. Studies by Hellström et al. [52] show that ELBW/ELGA neonates have low serum IGF-I at birth, thus predisposing them to development of severe retinopathy of prematurity. Data emerging from our laboratory suggest that IH may further compromise postnatal growth by abrogating factors that promote growth such as GH, IGF-I and VEGF [53]. The GH/IGF-I system, which consists of GH, GH receptor (GHR), IGF-I, IGF-II, IGF-I receptor (IGF-IR), and IGF binding proteins (IGFBPs 1–6), plays a critical role in fetal and postnatal growth and development [54,55]. GH is produced by the anterior pituitary gland and is an important regulator of linear growth, metabolism, and body composition from childhood to adult life [56]. It travels through the circulation to cells and target tissues that express its receptor [57], with the liver being its major target [58]. Once in the liver, GH promotes postnatal somatic growth by inducing the production of IGF-I in the liver, the main regulator of fetal and postnatal growth. IGF-I is mainly, but not exclusively, derived in the liver and is dependent on nutrient intake. Its availability is tightly regulated by its binding proteins (IGFBPs), which increase IGF-1 half-life from minutes to hours, and shuttles IGF-I to specific target tissues [59]. IGF-I is present in high concentrations in serum, and is mostly present as a ternary complex with IGFBP-3 and acid labile subunit (ALS) [60]. Free IGF-I represent only as a small percentage of total IGF-I. Approximately 90% of IGF-1 is bound to IGFBP-3, the primary hepatic-derived IGFBP, which serves as its major constitutive binding protein [61], although some IGF-I circulates in binary complexes with the various IGFBPs [62]. In the present study, CoQ10 supplementation in RA elevated serum GH levels. In 50–12% O_2_, serum GH was also elevated during olive oil and CoQ10 supplementation, whereas in 21–12% O_2_, serum GH was reduced with CoQ10 and significantly elevated with *n*-3 PUFA supplementation, demonstrating the differential responses of CoQ10 and *n*-3 PUFAs on growth factors when administered in IH. Low GH and GHR during CoQ10 exposure in 21–12% O_2_ did not alter somatic growth, but was associated with lower liver/body weight ratios. Similarly, higher serum GH and GHR with n-3 PUFAs in 21–12% O_2_ did not influence somatic growth, but resulted in smaller kidney weights compared to olive oil controls. In IHR post treatment, serum GH was lowest in the *n*-3 PUFA group compared to the other groups exposed to 21–12% O_2_ despite greater somatic growth. Instead, serum IGF-I levels were highest in the n-3 PUFA supplemented groups during treatment and the levels remained elevated during IHR post treatment, suggesting that *n*-3 PUFA-induced IGF-I accounts for the increased body weight and body length in those groups. Thus, it may be that during the perinatal period, GH does not play a similar important role in growth as does IGF-I [63], confirming the anabolic effects of IGF-I as a major stimulus of longitudinal growth [64]. The finding of higher IGF-I with *n*-3 PUFAs is corroborated by previous studies [65], despite major differences in species, age and oxygen status. IGFBP-3 was suppressed in RA with *n*-3 PUFAs, while in 21–12% O_2_, it was robustly increased with olive oil and decreased with both CoQ10 and *n*-3 PUFA supplementation. Reductions in serum IGFBP-3 were also reported in adults receiving *n*-3 PUFA supplementation [66]. Reductions in serum IGFBP-3 with elevated IGF-I would cause a decrease in IGF-I half-life, but will increase the transport of IGFs across the vascular endothelium and modulate the bioavailability of IGFs to target tissues [61]. IGFBP-1 is secreted by hepatocytes and blocks the actions of free, unbound IGF-I by preventing the binding of IGFs to their receptors [61]. Significant reductions in IGFBP-1 with *n*-3 PUFA supplementation may also participate in its growth promoting effects.

VEGF is a potent regulator of angiogenesis and vascular permeability factor. It is one of the most important molecules that governs the growth and differentiation of vascular smooth muscle and endothelium. Four major isoforms of human VEGF exist, namely, VEGF_121_, VEGF_165_, VEGF_189_, and VEGF_206_, (3 major isoforms in rodents, VEGF_120_, VEGF_164_ and VEGF_188_) from alternative splicing of a single gene [67], with VEGF_120_ and VEGF_164_ being soluble. VEGF signals to two major receptors, VEGFR-1 and VEGFR-2. Signaling of VEGF_165/164_ to VEGFR-2 results in potent angiogenesis. VEGF is highly regulated by oxygen tension and is rapidly induced by hypoxia and suppressed by hyperoxia. Unlike VEGFR-2, VEGFR-1 is also reactive to hypoxia [68]. VEGFR-1 is alternatively spliced to produce a soluble form that binds VEGF with high affinity. It is an endogenous negative regulator of VEGF which acts as a VEGF trap making it unavailable to its membrane receptor and thus decreasing VEGF angiogenesis action [69]. The present study shows that supplementation with CoQ10 or *n*-3 PUFAs had no appreciable effect on IH-induced VEGF during treatment, although the levels appeared to be lower with *n*-3 PUFAs. However, there was a latent suppression of VEGF with *n*-3 PUFA supplementation, but not with CoQ10, although the levels were not comparable with controls. This finding is important since IH-induced VEGF can result in damaged, leaky vessels. Resumption of blood flow through damaged vessels during re-oxygenation often results in reperfusion injury. Therefore, supplementation with *n*-3 PUFAs may lessen, but not eliminate the risk and severity of reperfusion injury. The responses of sVEGFR-1 mirrored those of VEGF in RA and IH regardless of treatment and provide further support for the response of VEGFR-1 to hypoxia. IGF-I is a permissive factor for VEGF induction [70]. Since IGF-I was induced with *n*-3 PUFA supplementation, it seems reasonable that VEGF would also be induced. The lack of total suppression of VEGF by *n*-3 PUFA supplementation may implicate an IGF-I influence.

### 4.3. Antioxidants

Another interesting finding of the current study included the effects of CoQ10 and *n*-3 PUFAs on antioxidants. During exposure to 50–12% O_2_, serum SOD was induced in the olive oil and *n*-3 PUFA groups, but was effectively reduced with CoQ10, an effect that lasted during the IHR period. The principal ROS is a superoxide anion which is rapidly dismutated to H_2_O_2_ and O_2_ by mitochondrial, cytosolic, and peroxisomal SOD, the primary ROS detoxifying enzymes. SOD is induced or suppressed to match superoxide anion production, such that a more superoxide anion leads to more SOD, and more SOD leads to more H_2_O_2_. Therefore, reduction of SOD in IH with CoQ10 suggests curtailed ROS and less H_2_O_2_ production. This effect of CoQ10 was not surprising as there are numerous reports of the benefits of CoQ10 on reduction of oxidative stress. Since CoQ10 is fat soluble, it is readily absorbed and protects against lipid peroxidation in tissues with high lipid content such as the brain and liver which are targets for lipid peroxidation (a self-propagating chain reaction that involves H_2_O_2_ reacting with free iron to form the hydroxyl radical) and IH injury. Interestingly, *n*-3 PUFAs also reduced SOD levels but only during the IHR period, and only in the RA and 21–12% O_2_ groups, suggesting latent or weak effects on ROS. The cell uses glutathione and catalase to catalyze the decomposition of H_2_O_2_ into water and O_2_. In addition to SOD reduction, there was a robust increase in catalase with CoQ10 which may provide further evidence of effective ROS scavenging, particularly H_2_O_2_. We have previously shown that 50–12% O_2_ models result in H_2_O_2_ accumulation [6]. As a corollary, we now provide evidence that CoQ10 supplementation induces catalase to curtail H_2_O_2_ accumulation in IH. Exposure to 21–12% O_2_ also resulted in elevated catalase activity in the olive oil and *n*-3 PUFA groups, concurrent with elevated SOD activity. This may indicate continued propagation of H_2_O_2_. Sustained induction of catalase during IHR post treatment suggests that CoQ10 results in an immediate and sustained increase in catalase in all O_2_ conditions, while treatment with *n*-3 PUFA in RA results in a latent elevation during IHR. Glutathione levels were highly induced with 21–12% O_2_ during oil, and to a lesser degree during *n*-3 PUFA treatment and were not appreciably elevated during IHR. *n*-3 PUFAs appeared to be most effective for inducing glutathione in RA and 21–12% O_2_ during IHR. While glutathione is also involved in H_2_O_2_ scavenging predominantly in the mitochondria, catalase is best with high concentrations of H_2_O_2_ [71]. Taken together, these findings suggest that CoQ10 supplementation is very effective for scavenging IH-induced ROS and may protect against “oxygen radical diseases of the neonate”. 

## 5. Conclusions

We have shown for the first time that regardless of oxygen tension resolution between IH events, neonatal IH causes significant deleterious effects on somatic growth. Supplementation with CoQ10 during IH may curtail ROS accumulation by inducing antioxidant activities, while supplementation with *n*-3 PUFAs has beneficial effects on growth and growth promoting factors, and may even provide moderate protection against IH-induced vascular damage by reducing VEGF levels. Although we assessed differences in the organ/body weight ratios, we did not employ histological methods to assess pathology or to measure cell size and cell number in the organs of interest. However, this limitation does not diminish the importance of the findings as it relates to the possible benefits of *n*-3 PUFA and/or CoQ10 supplementation in ELBW/ELGA neonates at risk for IH-induced growth restriction and oxidative stress. Further studies are needed to assess the synergistic benefits of CoQ10 and *n*-3 PUFA co-administration during neonatal IH. 

## Figures and Tables

**Table 1:** Organ Weight at P14.

	Olive Oil	CoQ10	*n*-3 PUFAs
**RA**			
Brain/Body Wt. Ratios	0.05 ± 0.0063	0.05 ± 0.010	0.06 ± 0.01 *
Heart/Body Wt. Ratios	0.006 ± 0.006	0.007 ± 0.002	0.007 ± 0.0017
Lung/Body Wt. Ratios	0.02 ± 0.003	0.02 ± 0.0045	0.02 ± 0.0034
Liver/Body Wt. Ratios	0.03 ± 0.003	0.028 ± 0.0064 *	0.03 ± 0.0017
Kidney/Body Wt. Ratios	0.013 ± 0.0018	0.015 ± 0.0032	0.015 ± 0.003 *
**50–12% O_2_ IH**			
Brain/Body Wt. Ratios	0.05 ± 0.011	0.06 ± 0.0056 *	0.05 ± 0.0054
Heart/Body Wt. Ratios	0.006 ± 0.0012	0.007 ± 0.0015 *	0.007 ± 0.0095 *
Lung/Body Wt. Ratios	0.02 ± 0.0034	0.02 ± 0.0018	0.02 ± 0.0016
Liver/Body Wt. Ratios	0.025 ± 0.0026	0.02 ± 0.0033 *	0.03 ± 0.0044
Kidney/Body Wt. Ratios	0.014 ± 0.0027	0.013 ± 0.002 *	0.014 ± 0.0022
**21–12% O_2_ IH**			
Brain/Body Wt. Ratios	0.05 ± 0.0071	0.05 ± 0.005	0.05 ± 0.006
Heart/Body Wt. Ratios	0.007 ± 0.0015 *	0.006 ± 0.0012	0.006 ± 0.0009
Lung/Body Wt. Ratios	0.02 ± 0.0042	0.02 ± 0.0032	0.019 ± 0.0025
Liver/Body Wt. Ratios	0.03 ± 0.0049	0.025 ± 0.0036 *	0.028 ± 0.0047
Kidney/Body Wt. Ratios	0.015 ± 0.0033	0.014 ± 0.0024	0.013 ± 0.0016*

*n* = 18 rats/group. Data are mean ± SD (* *p* < 0.05 compared to olive oil). PUFAs: polyunsaturated fatty acids; RA: room air; IH: intermittent hypoxia.

**Table 2:** Organ Weight at P21.

	Olive Oil	CoQ10	*n*-3 PUFAs
**RA**			
Brain/Body Wt. Ratios	0.035 ± 0.0035	0.036 ± 0.0053	0.039 ± 0.0044 **
Heart/Body Wt. Ratios	0.007 ± 0.0016	0.007 ± 0.0014	0.006 ± 0.0008
Lung/Body Wt. Ratios	0.01 ± 0.0017	0.01 ± 0.0012	0.01 ± 0.0026
Liver/Body Wt. Ratios	0.04 ± 0.0061	0.05 ± 0.0081 **	0.047 ± 0.0083 *
Kidney/Body Wt. Ratios	0.013 ± 0.0016	0.014 ± 0.0029	0.015 ± 0.002 *
**50–12% O_2_ IHR**			
Brain/Body Wt. Ratios	0.046 ± 0.0082	0.058 ± 0.0051 **	0.05 ± 0.0042
Heart/Body Wt. Ratios	0.006 ± 0.0009	0.006 ± 0.0008	0.006 ± 0.0012
Lung/Body Wt. Ratios	0.01 ± 0.0025	0.01 ± 0.0018	0.01 ± 0.0021
Liver/Body Wt. Ratios	0.03 ± 0.0061	0.03 ± 0.0056	0.04 ± 0.0072 **
Kidney/Body Wt. Ratios	0.012 ± 0.0025	0.011 ± 0.0023	0.012 ± 0.0023
**21–12% O_2_ IHR**			
Brain/Body Wt. Ratios	0.04 ± 0.0062	0.039 ± 0.004	0.03 ± 0.0038
Heart/Body Wt. Ratios	0.006 ± 0.0009	0.0057 ± 0.0009	0.006 ± 0.0009
Lung/Body Wt. Ratios	0.01 ± 0.003	0.01 ± 0.0012	0.01 ± 0.0019
Liver/Body Wt. Ratios	0.03 ± 0.007	0.025 ± 0.0058 *	0.039 ± 0.0054 **
Kidney/Body Wt. Ratios	0.012 ± 0.002	0.013 ± 0.0018	0.012 ± 0.0017

*n* = 18 rats/group. Data are mean ± SD (* *p* <0.05; ** *p* < 0.01 compared to olive oil). IHR: recovery from IH).
